# Supplementary material for: Seasonal changes in the abundance and biomass of copepods in the south-eastern Baltic Sea in 2010 and 2011
Source: PeerJ. 2018 Sep 6;6:e5562. doi: 10.7717/peerj.5562 (PMC6132220; doi:10.7717/peerj.5562)
Supplement: Table S1 [file peerj-06-5562-s001.docx]

| Data | Time of sampling | Layer [m] | | |
| --- | --- | --- | --- | --- |
|  |  | Bottom-upper limit of halocline | Upper limit of halocline-upper limit of thermocline | Upper limit of thermocline-surface |
| 11.02.10  (winter 2010) | 8:00 | 102 – 90 | 90 – 60 | 60 – 0 |
| 18.04.10  (spring 2010) | 10:40 | 100 – 65 | 65 – 25 | 25 – 0 |
| 03.06.10  (summer 2010) | 1:00 | 90 – 30 | 30 – 10 | 10 – 0 |
| 31.03.11  (winter 2011) | 4:00 | 100 – 85 | 85 – 65 | 65 – 0 |
| 07.05.11  (spring 2011) | 21:45 | 100 – 70 | 70 – 25 | 25 – 0 |
| 11.06.11  (summer 2011) | 14:40 | 100 – 70 | 70 – 25 | 25 – 0 |
| 12.11.11  (autumn 2011) | 8:00 | 100 – 50 | 50 – 25 | 25 – 0 |
