# Supplementary material for: Seasonal changes in the abundance and biomass of copepods in the south-eastern Baltic Sea in 2010 and 2011
Source: PeerJ. 2018 Sep 6;6:e5562. doi: 10.7717/peerj.5562 (PMC6132220; doi:10.7717/peerj.5562)
Supplement: Table S2 [file peerj-06-5562-s002.docx]

| Data | Layer [m] | | | |
| --- | --- | --- | --- | --- |
|  | 10 – 0 | 20 – 10 | 30 – 20 | 40 – 30 |
| 10 March 2010 | + | + | + | + |
| 01 April 2010 | + | + | + | + |
| 24 May 2010 | + | + | + | + |
| 21 June 2010 | + | + | + | + |
| 27 July 2010 | + | + | + | + |
| 19 August 2010 | + | + | + | + |
| 14 Sept. 2010 | + | + | + | + |
| 13 Oct. 2010 | + | + | + | + |
| 19 Nov. 2010 | + | + | + | + |
| 14 Jan. 2011 | + | + | + | + |
| 05 April 2011 | + | + | + | + |
| 16 May 2011 | + | + | + | + |
| 03 June 2011 | + | + | + | + |
| 27 June 2011  (collected instead of July) | + (20 – 0) | | + | + |
| 03 Sept. 2011  (collected instead of August) | + | + | + | + |
| 26 Sept. 2011 | + | + | + | + |
| 28 Oct. 2011 | + | + | + | + |
| 29 Nov. 2011 | + | + | + | + |
