# Supplementary figures and images for: Seasonal changes in the abundance and biomass of copepods in the south-eastern Baltic Sea in 2010 and 2011
Source: PeerJ. 2018 Sep 6;6:e5562. doi: 10.7717/peerj.5562 (PMC6132220; doi:10.7717/peerj.5562)

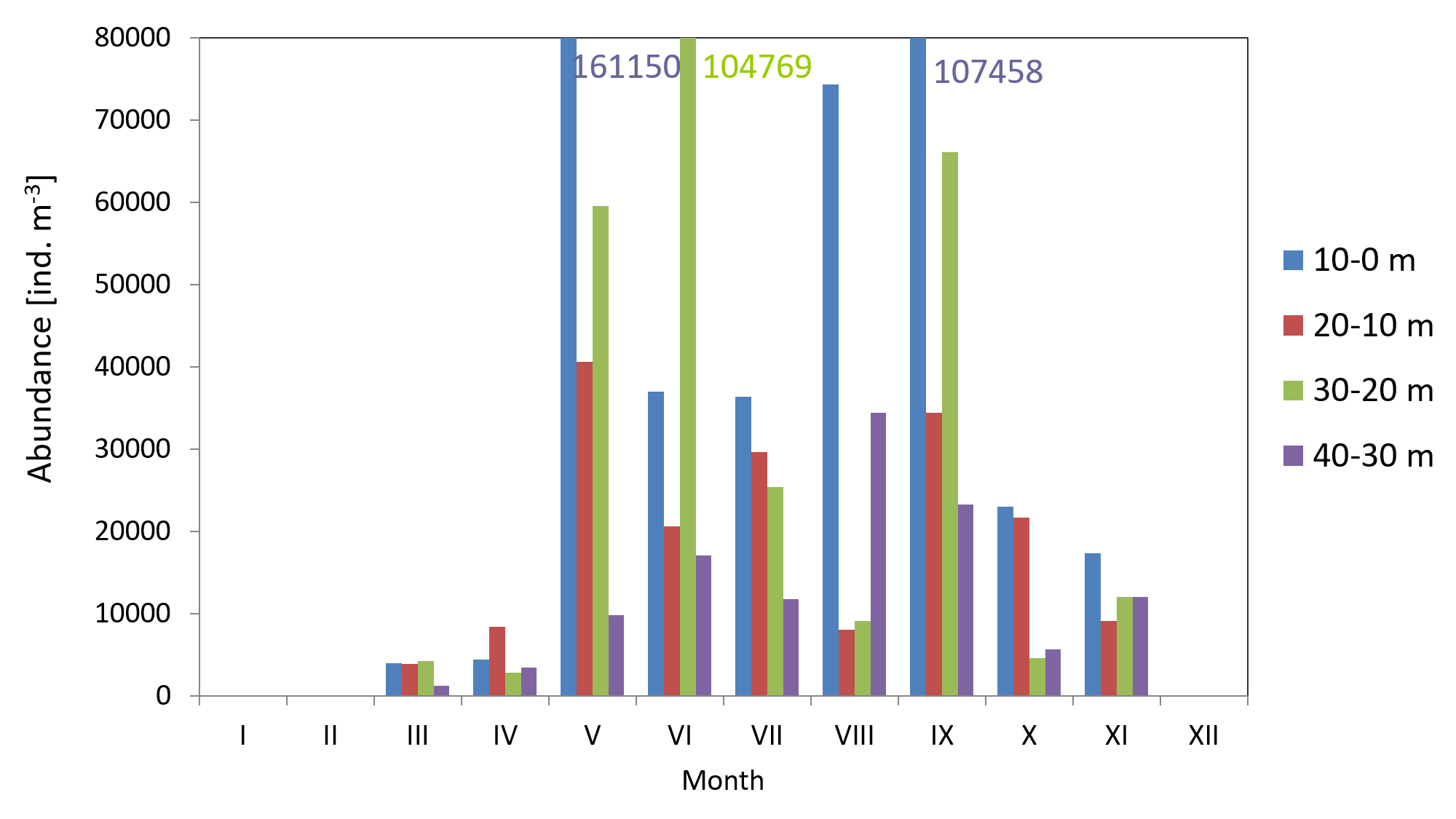

Supplement: Figure S1 [file peerj-06-5562-s003.png]

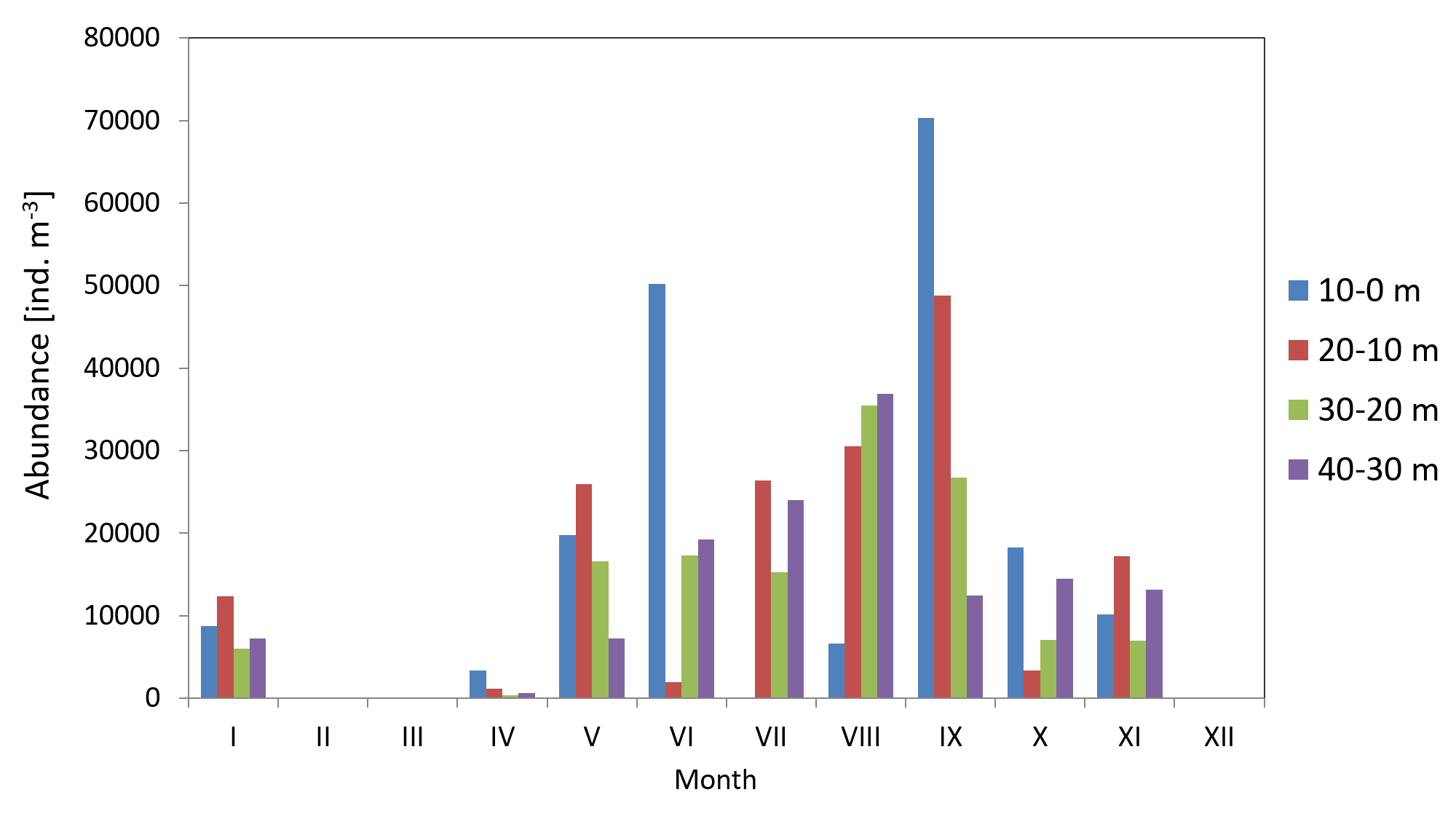

Supplement: Figure S2 [file peerj-06-5562-s004.png]

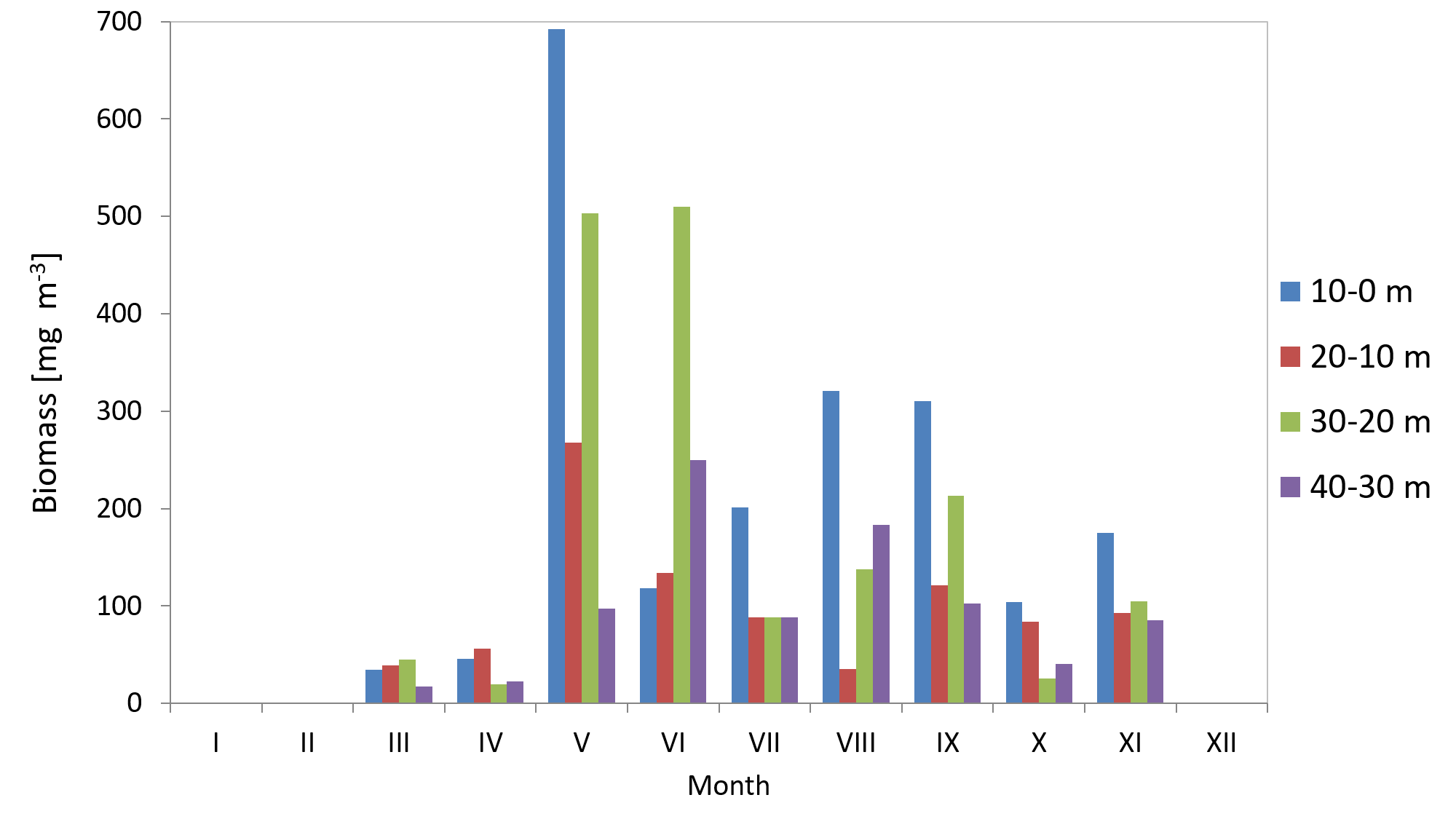

Supplement: Figure S3 [file peerj-06-5562-s005.png]

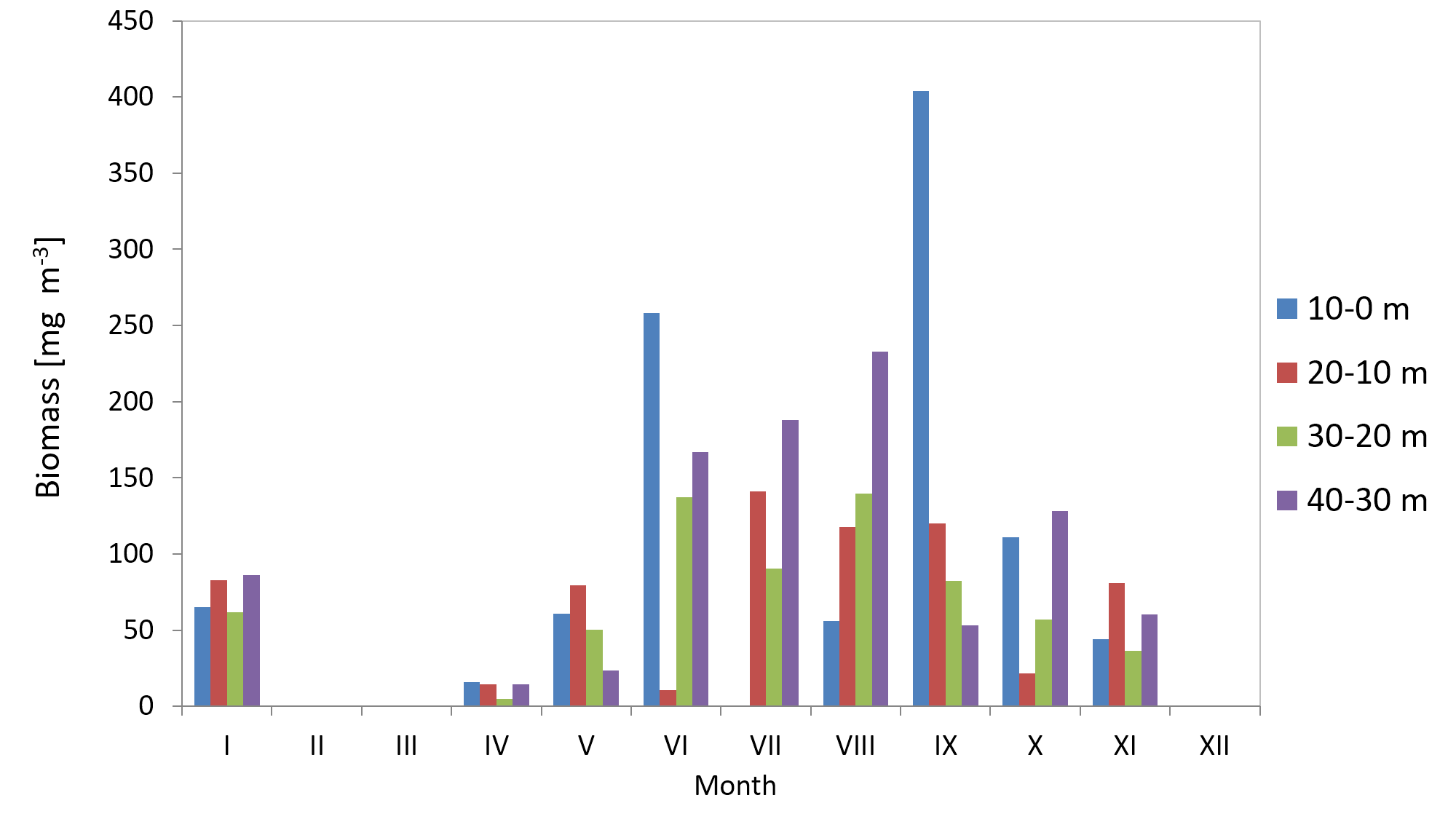

Supplement: Figure S4 [file peerj-06-5562-s006.png]
